# Beyond Starch: Towards a Scalable Potato Platform for Molecular Farming

**DOI:** 10.1111/pbi.70616

**Published:** 2026-03-29

**Authors:** Izabela Anna Chincinska, Dorota Sołtys‐Kalina, Audrey Y.‐H. Teh

**Affiliations:** ^1^ Department of Plant Experimental Biology and Biotechnology, Laboratory of Plant Biotechnology, Faculty of Biology University of Gdańsk Poland; ^2^ Plant Breeding and Acclimatization Institute – National Research Institute in Radzików Młochów Division Poland; ^3^ City St George's, University of London, Institute for Infection & Immunity Department of Medicine, Jenner Wing London UK; ^4^ Centre for Research in Biotechnology for Agriculture (CEBAR) University of Malaya Kuala Lumpur Malaysia

**Keywords:** bioreactor potato, genome editing, glycoengineering, plant molecular farming, protein storage vacuole, recombinant proteins, *Solanum tuberosum*, technoeconomic analysis

## Abstract

Thirty‐five years after the first recombinant protein was produced in potato and 30 years after clinical trials of edible vaccines from its tubers, the crop is being reconsidered as a molecular farming chassis. Potatoes can accumulate recombinant proteins in tubers, enabling long‐term storage and simplified logistics. Clonal propagation, access to minitubers and microtubers, and an established production infrastructure further distinguish the platform. Limited pollen dispersal and reliance on vegetative propagation also provide biosafety advantages. Despite these features, potato lost ground to other hosts due to low expression levels, high downstream processing (DSP) costs in water‐ and starch‐rich tissues and limited scalability relative to seed‐propagated crops. We review the technical and economic factors behind this decline and assess new opportunities to overcome them. Recent advances include refined expression cassettes, ER and secretory pathway engineering (including targeted glycoengineering), multigene stacking, genome editing and enzyme‐assisted DSP. Seed‐propagated diploid potatoes and alternative *Solanum* germplasm offer additional chassis options. Together with the emergence of start‐ups revisiting potato as a production platform, these advances point to practical routes for re‐evaluating its role in plant molecular farming. We argue that a rationally engineered, diploid‐based ‘bioreactor potato’ could complement existing hosts and re‐establish relevance in specific niches of next‐generation biomanufacturing.

## Graphical abstract

Re‐engineering potato as a biosafe and host‐optimised platform for plant molecular farming by integrating intrinsic biological traits with targeted engineering strategies.

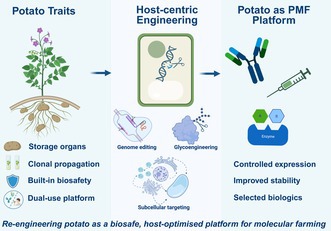

**Abbreviations**
16DOX: 16α‐hydroxylase
CAPEX: Capital expenditure
CHO: Chinese hamster ovary
DSP: Downstream processing
DW: Dry weight
ER: Endoplasmic reticulum
FW: Fresh weight
GLA: Glycoalkaloid
GT: Glycosyltransferase
HSA: Human serum albumin
IgG: Immunoglobulin G
mAb: Monoclonal antibody
NGTs: New genomic techniques
ORF: Open reading frame
PMF: Plant molecular farming
PSV: Protein storage vacuole
tPINII: Proteinase inhibitor II terminator
RPs: Recombinant proteins
SP: Signal peptide
TSP: Total soluble protein
UAV: Unmanned Aerial Vehicle
VLP: Virus‐like particle

## 1 Introduction

More than 3 decades after the first demonstration of a human protein produced in potato ( *Solanum tuberosum* L.) (Sijmons et al. 1990), the approach of using plants as biofactories has evolved from proof‐of‐concept to an increasingly engineered discipline. Early studies quickly recognised potato as a promising chassis for plant molecular farming (PMF). Although initial experiments targeted leaves, tubers soon proved more attractive as a production organ because they can accumulate and store proteins for extended periods. Soon after, it was also demonstrated that transgenic tubers producing antigens could be used as edible vaccines, inducing immune responses following oral delivery (Haq et al. 1995; Mason et al. 1998; Tacket et al. 1998). By the late 1990s and early 2000s, a broad spectrum of recombinant proteins (RPs) had been reported in potato, including cytokines, viral antigens, antibodies and even spider silk proteins (Artsaenko et al. 1998; Chen and Liu 2011; Farran et al. 2002; Scheller et al. 2001).

Beyond these early demonstrations, potato also shares the general advantages attributed to plant‐based expression systems. Plants are inherently safe for the production of biologics, as they are free from human pathogens, oncogenic sequences and endotoxins (Buyel 2023). They can perform complex post‐translational modifications, including glycosylation, which are essential for protein solubility, stability and pharmacological function (Göritzer et al. 2022; Yehuda and Padler‐Karavani 2020). At the same time, cultivation requires comparatively low capital expenditure (CAPEX) and enables flexible scalability without the need for costly fermenters (Holtz et al. 2015; McDonald and Holtz 2020). These collective features have long positioned plants as attractive, versatile and potentially cost‐effective alternatives to microbial and mammalian production systems.

Despite these prospects, PMF as a whole struggled to achieve commercial success. Microbial systems such as *Escherichia coli* and mammalian cell cultures, notably Chinese hamster ovary (CHO) cells, became dominant in biopharmaceutical manufacturing (Buyel 2026). Plant‐derived products remained few, and scepticism grew over their industrial relevance (Buyel 2024; Schillberg et al. 2019). One notable exception was the approval of recombinant glucocerebrosidase from carrot cell cultures (*Daucus carota* L.) for the treatment of Gaucher's disease (Shaaltiel et al. 2007), yet such successes remained isolated. Within this broader decline, potato was particularly affected. Once a frontrunner in the 1990s, its progress was slowed by low expression levels in tubers, the high downstream processing (DSP) burden associated with water‐ and starch‐rich tissues, and comparatively limited scalability relative to seed‐propagated crops. As other plant hosts advanced, especially *Nicotiana benthamiana*, rice ( *Oryza sativa* L.) and maize ( *Zea mays* L.), potato's role in PMF diminished even further.

**FIGURE 1:**
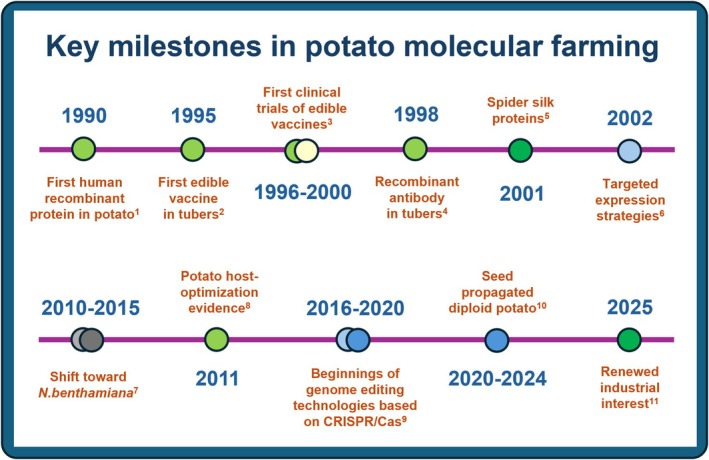
Key milestones in potato PMF. Explanations of milestones indicated on the timeline: ^1^Human serum albumin (HSA) expressed in leaves (Sijmons et al. 1990), ^2^First edible vaccine: *E. coli* LT‐B antigen expressed in tubers (Haq et al. 1995), ^3^First clinical trials of edible vaccines: NVCP and HBsAg tested in humans (Mason et al. 1996; Tacket et al. 1998, 2000),^4^Antibodies demonstrated in potato tubers (Artsaenko et al. 1998), ^5^Spider silk proteins (spidroins) expressed in potato (Scheller et al. 2001), ^6^Targeted expression strategies directing RPs to tubers and subcellular compartments (Farran et al. 2002), ^7^Shift toward *Nicotiana benthamiana* as the dominant PMF host (Buyel 2024, 2026; Göritzer, Kallolimath, and Strasser 2025), ^8^First host‐optimisation evidence: ATP/ADP transporter knockdown increased RP yield in tubers (Tremblay, Diao, et al. 2011), ^9^First CRISPR/Cas reports appeared, later developed for breeding and research (reviewed in Chincinska et al. 2023), ^10^Seed‐propagated diploid potato adopted in breeding programmes (Bethke et al. 2022; Xin et al. 2025), ^11^Renewed industrial interest: Biotech start‐ups (e.g., PoLoPo, Israel) announce projects exploring potato for PMF, highlighting growing interest beyond academia (Turrell 2025).

In the past few years, however, plant molecular farming has been regaining momentum. Recent clinical trials with plant‐derived vaccines, including candidates against influenza and SARS‐CoV‐2, have convincingly demonstrated that plant systems can deliver safe, immunogenic and effective biologics (Fausther‐Bovendo and Kobinger 2021; Hager et al. 2022). At the same time, rapid progress in glycoengineering and endoplasmic reticulum (ER) pathway engineering has markedly improved the quality and functionality of complex recombinant proteins, particularly antibodies (Göritzer et al. 2022, 2024; Göritzer, Melnik, et al. 2025; Strasser 2023). Complemented by increasingly precise technoeconomic analyses that chart strategies to overcome remaining bottlenecks (Buyel 2026; Ridgley et al. 2023), these advances provide a strong rationale for re‐evaluating plant systems in next‐generation biomanufacturing. In this context, it is pertinent to ask what role potato might play. With breakthroughs in potato genetics and biotechnology, ranging from CRISPR/Cas‐mediated editing to diploid, seed‐propagated germplasm, the crop is once again poised for reconsideration. Notably, this renewed attention is not limited to academic advances. Commercial interest in potato as a biofactory chassis is also emerging. For example, recent biotech start‐ups (e.g., PoLoPo, Israel; Turrell 2025) have announced initiatives to develop potato lines for molecular farming. Although most of the available information originates from company communications rather than peer‐reviewed studies, such developments illustrate that potato is beginning to attract attention beyond academia. Key milestones in the development of potato as a PMF platform are summarised in Figure 1.

## 2 Potato in Molecular Farming

Potato offers a versatile platform for PMF due to its well‐established agricultural infrastructure and widespread cultivation (Chincinska et al. 2023). Tubers provide a naturally protective environment for RPs, allowing long‐term storage with partial retention of activity (Artsaenko et al. 1998; De Wilde et al. 2002). A further advantage is the relative ease of establishing stable transgenic lines, facilitated by vegetative propagation and clonal maintenance, which allows faster deployment compared to seed‐propagated crops such as tobacco (De Wilde et al. 2002; Gharghi et al. 2023). While tubers can accumulate RPs (Table 1), actual yields are generally modest, highlighting the need for optimised expression strategies. Nevertheless, the storage capacity and robustness of tubers remain advantageous for applications in regions with limited infrastructure. However, large‐scale expansion remains more logistically demanding than in seed‐propagated crops.

**TABLE 1:** Production of recombinant proteins in potato.

Protein class/application	Protein (abbreviation, full name, MW [kDa])	Function/Application	Plant material/transformation system	Expression cassette and targeting	Tissue produced RP/Maximal yield^a^	References
Vaccine antigens and immunogens	LT‐B – *E. coli* heat‐labile enterotoxin B subunit (11.6 kDa)	B subunit of *E. coli* heat‐labile enterotoxin/oral vaccine	cv. Frito‐Lay 1607/microtubers	p35S (double enhancer) or pB33, TEV‐5′UTR/SEKDEL, 3′VSP SP/ER retention	Microtubers/110 μg·g‐1 TSP	Haq et al. 1995
			cv. Frito‐Lay 1607/LBA4404, leaf discs	codon‐optimised synthetic CDS, p35S (double enhancer), TEV‐5′UTR/native LT‐B SP 3′VSP/ER retention	Tubers:1.92 μg·mg‐1 TSP (3.7–15.7 μg·g‐1 FW)	Mason et al. 1998; Tacket et al. 1998
	HBsAg – Hepatitis B surface antigen (24 kDa)	Envelope protein of human hepatitis B virus/proof‐of‐concept expression in potato	cv. Nevsky/GV3850/minitubers	p35S, 3′NOS/ND	Leaves: 3–11 ng·mg‐1 TSP; Roots: 76–83 ng·mg‐1 TSP	Domansky et al. 1995
		Envelope protein of human hepatitis B virus/oral vaccine	cv. Frito‐Lay 1607/LBA4404/microtubers	(a)p35S (double enhancer), TEV‐5′UTR, VSP‐3′UTR/native HBV signal peptide/Apoplast (b)p35S (double enhancer), TEV‐5′UTR, VSP‐3′UTR, tPINII/VSP‐aS or VSP‐aL SP (~SEKDEL)/ER retention	(a)Leaves: 0.29% TSP Tubers: 6.5–8.5 μg·g‐1 FW (b)Leaves: 0.25% TSP Tubers:16 μg·g‐1 FW	Richter et al. 2000 Thanavala et al. 2005
		Envelope protein of human hepatitis B virus/proof‐of‐concept expression in potato hairy roots	cv. Kufri Bahar/EHA105/internodal stems/A. rhizogenes ATCC1583/internodal segments (hairy roots induction)	pEFE, tNOS/SEKDEL/ER retention	Plant: 19.11 ng·g‐1 FW; Microtubers: 23.94 ng·g‐1 FW; Hairy roots: 97.10 ng·g‐1 FW	Kumar et al. 2006
	HBsAg fragment – Hepatitis B surface antigen fragment (16–17 kDa)	Envelope protein of human hepatitis B virus/model HBV surface antigen fragment	ND/GV3850/microtuber discs	p35S (double enhancer) or pPATATINE ± 5′TEV, t35S/Cytosol	Tubers: 0.05% TSP	Bondarkhilli et al. 2024
	VP60 – Capsid virus protein VP60 (60 kDa)	Structural protein of rabbit hemorrhagic disease (RHDV)/vaccine	cv. Désirée/LBA4404/leaf explants	p35S or p35S (double enhancer)/VLPs in cytosol	Leaves/2.97 μg·mg‐1 TSP Microtubers/702 ng·mg‐1 TSP	Castañón et al. 1999
	GP5 – Glycoprotein 5 (25 kDa)	Envelope glycoprotein of porcine reproductive and respiratory syndrome virus (PRRSV) of swine/oral vaccine	cv. E‐potato 3 (E3)/LBA4404/microtubers	p35S/native GP5 signal peptide/ER retention	Leaves: 2.5–4.7 μg·g‐1 TSP Tubers: 0.8–1.2 μg·g‐1 TSP	Chen and Liu 2011
	Aβ42 – β‐amyloid peptide (4 kDa)	Component of amyloid plaques in the brain, Alzheimer’s disease/Model antigen for experimental Alzheimer’s immunotherapy	cv. Désirée/LBA4404/leaf explants	p35S, 5′TEV, t35S/Cytosol	ND	Kim et al. 2003
				p35S, 5′TEV, tandem Aβ1–42 repeats, SEKDEL, t35S/ER retention	ND	Youm et al. 2005
Human functional proteins	HSA – Human serum albumin (66.5 kDa)	Serum protein/therapeutic use candidate/proof‐of‐concept expression in potato	cv. Désirée/ND/tuber discs	p35S double enhancer, AMV leader/Apoplast	Leaves: 0.02% TSP	Sijmons et al. 1990
			cv. Désirée, cv. Kennebec, Pas58/GV2260/leaf explants	pB33/PINII‐SP/Apoplast	Tubers: 0.1%–0.2% TSP	Farran et al. 2002
	hEGF – Human epidermal growth factor (6 kDa)	Peptide hormone for epidermal regeneration/potential application in wound healing	cv. Nevsky/GV3850/minitubers	p35S, pB33/Cytosol	Roots: ~150 pg·mg^−1^ TSP; Tubers: ~170 pg·mg^−1^ TSP	Salmanian et al. 1996
	Hbca – β‐casein (30 kDa)	Nutritional protein for infants/proof‐of‐concept expression in potato	cv. Bintje/GV3101 (pMP90RK)/leaf discs	mas P19, mas P29 promoters; 3′g7pA/Cytosol	Leaves: 0.01% TSP	Chong et al. 1997
	hLF – Human lactoferrin (80 kDa)	Antimicrobial and immunoregulatory protein/proof‐of‐concept expression in potato	cv. Bintje/GV3101 (pMP90RK)/leaf discs	(a) p35S tandem promoter, SEKDEL, 3′NOS/ER retention (b) mas P2 promoter, SEKDEL, 3′g7pA/ER retention	Tubers: ~0.01% TSP (35S); up to ~0.1% TSP (mas P2, auxin‐induced)	Chong and Langridge 2000
	IL‐2 – Interleukin‐2 (15–16 kDa)	Cytokine stimulating T and NK cells/proof‐of‐concept expression in potato	cv. Jopung/AGL1/stem explants	(a) p35S; (b) B33; 3′NOS/ND	Microtubers: 49–115 U·g^−1^ FW	Park and Cheong 2002
	hCT – Human calcitonin (13 kDa)	Peptide hormone regulating Ca and P metabolism/proof‐of‐concept	cv. Nevsky/GV3850/minituber (tuber) discs	p35S/TEV ‐ 5′UTR/Cytosol	Leaves: ~2.0 ± 0.3 ng·mg^−1^ TSP Roots: ~1.0 ± 0.2 ng·mg^−1^ TSP Tubers: ~0.7 ± 0.2 ng·mg^−1^ TSP	Ofoghi et al. 1999; 2000; 2004; 2012
	hCT – Human calcitonin (13 kDa)	Peptide hormone regulating Ca and P metabolism/proof‐of‐concept	cv. Kardal, cv. Marfona/LBA4404/leaf and tuber discs	pB33 vs p35S/Cytosol	Tubers (cv.Kardal, pB33): ~1.8 ng·mg^−1^ TSP Tubers (cv.Kardal, p 35S): ~0.7 ng·mg^−1^ TSP	Ghorbaniparsa and Ofoghi 2016
Antibodies	scFv – (anti‐β‐1,4‐endoglucanase) (32 kDa)	Antibody fragment – antigen binding domain/proof‐of‐concept, pathogen‐targeting	Dihaploid 6487‐9/AGL0/internodal stem explants	p35S; VH–VL joined by linker peptide (202′ or (G_4_S)_3_); ±KDEL/KDEI; no SP/Cytosol	Roots: ~0.03% TSP (−KDEL); 0.15%–0.3% TSP (+KDEL or KDEI)	Schouten et al. 1997
	scFv – (anti‐hapten 2‐phenyl‐oxazolone) (~30–32 kDa)	Antibody fragment – antigen binding domain/proof‐of‐concept, pathogen‐targeting	cv. Désirée/ *A. tumefaciens* /leaf explants	p35S; LeB4‐SP; c‐myc‐tag; KDEL/ER retention	Up to ~2% of TSP (typical lines 0.5%–1% TSP; ~70ng scFv/80μg TSP)	Artsaenko et al. 1998
	IgG – Immunoglobulin G (MAK33) (50 kDa)	Model murine antibody (IgG and Fab fragments)/testing expression and stability – proof‐of‐concept	cv. Désirée/GV2260/root nodal segments	(a) p35S, 5′2S2‐SP, tOCS/ER retention (b) p35S, 5′2S2‐SP, 3′DIKDEL, tE9/Apoplast	(a) ER retention: Leaves: IgG 0.75% TSP; Fab 3.9% TSP Tubers: IgG 0.55% TSP; Fab 0.46% TSP (b) Apoplast: Leaves: IgG 0.24% TSP; Fab 0.20% TSP Tubers: IgG 0.13% TSP; Fab 0.15% TSP	De Wilde et al. 2002
	scFv L1G6 – (human monoclonal antibody fragment; ~30–32 kDa)	Evaluation of an improved potato bioreactor line	cv. Désirée (wild type and riAATP1‐10 RNAi line)/Agrobacterium tumefaciens (binary vector pMB 5–61)/tuber disc transformation (mini‐tubers)	p35S; c‐myc‐tag; KDEL/ER retention	Tubers: 0.5% TSP	Tremblay, Diao, et al. 2011
Enzymes	ansB – *E.coli* L‐asparaginase II (40.2 kDa)	Therapeutic enzyme used in acute lymphoblastic leukemia treatment/proof‐of‐concept expression in potato hairy roots	cv. Agria/ *A. rhizogenes* ATCC 15834/internodal segments (hairy roots induction)	p35S; Kozak + ATG, 6×His (N‐terminus); 3′KDEL/ER retention	Enzyme activity: 519,700 IU/ml	Mohammadi et al. 2020
Structural/biomaterial proteins	FA2, SD1, SM12, SO1, SO1SO1 – synthetic *Nephila clavipes* MaSp1 spidroin homologs (12.9–99.8 kDa)	Biomaterial applications, e.g., fibers, scaffolds, technical and medical materials	cv. Solaria, cv. Desi/ *A. tumefaciens* /leaf disc explants	p35S; LeB4‐SP; c‐myc‐tag; KDEL; t35S/ER retention	Leaves: 0.5%–1.0% TSP	Scheller et al. 2001

Abbreviations: (G_4_S)_3_ linker, canonical flexible linker composed of three repeats of the pentapeptide Gly_4_Ser [(Gly‐Gly‐Gly‐Gly‐Ser)_3_], commonly used to connect VH and VL in scFv constructs; 3′g7pA, polyadenylation signal of *A.tumefaciens* T‐DNA gene 7; used as a transcription terminator in plant expression constructs; AMV leader, 5′ untranslated leader sequence from RNA 4 of Alfalfa mosaic virus (functions as a translational enhancer in plant expression systems); CDS, coding sequence; ER, endoplasmic reticulum; FW, fresh weight; LeB4 SP, *Vicia faba* legumin B4 SP; linker peptide (202), short synthetic amino acid sequence derived from the original 202’scFv construct provides flexibility between VH and VL domains; mas P19/P29, mannopine synthase promoters ( *Agrobacterium tumefaciens* T‐DNA), bidirectional promoters regulating expression in plants; ND, not determined (not available); pEFE, promoter of banana ethylene forming enzyme gene (organ‐/tissue‐specific promoter); scFv, Single‐chain variable fragment; SP, signal peptide; t35S, terminator derived from the cauliflower mosaic virus 35S gene; tE9, terminator derived from the small subunit of ribulose‐1,5‐bisphosphate carboxylase/oxygenase (rbcS‐E9) gene of *Arabidopsis thaliana* ; tNOS, terminator from the nopaline synthase (NOS) gene of *Agrobacterium tumefaciens* ; tOCS, terminator from the octopine synthase (OCS) gene of *Agrobacterium tumefaciens* ; tPINII, terminator from the potato proteinase inhibitor II (PINII) gene; TSP, total soluble protein; U·g^−1^ FW, enzyme activity units per gram fresh weight; UTR, untranslated region; VH, variable domain of the heavy chain of an antibody; VL, variable domain of the light chain of an antibody; VLPs, virus‐like particles.

^a^ Yields are presented in the same units as reported by the original authors, without conversion or standardisation.

### 2.1 Tubers as Natural Biofactories for RPs

Potato stands out as a PMF platform due to its ability to produce significant biomass, of which up to 85% is located in tubers (Bradshaw 2021; Halterman et al. 2016). Tubers are modified underground stems that function as storage organs, accumulating starch as well as substantial amounts of protein. With a protein content of around 10% on a dry weight (DW) basis and about 2% on a fresh weight (FW), potato tubers rival cereals such as rice and wheat *(Triticum aestivum* L.*)*. Their protein content is largely made up of patatins and protease inhibitors (Bártová et al. 2015; Bradshaw 2021). Storage proteins in potato tubers are deposited primarily in protein storage vacuoles (PSVs), suggesting that this compartment could, in principle, be explored for RPs deposition in potato (Jørgensen et al. 2011; Marin Viegas et al. 2017; Ocampo et al. 2016).

Given this storage biology, tubers can be deployed in two complementary roles: production organs for purified RPs and edible tissues for direct delivery (Gupta et al. 2022). Early proof‐of‐concept studies in potato demonstrated mucosal and/or systemic immune responses to orally delivered antigens, including the *E. coli* heat‐labile toxin B subunit (LT‐B) in animals and human volunteers, the Norwalk virus capsid protein (NVCP) in humans, and the hepatitis B surface antigen (HBsAg) (Haq et al. 1995; Mason et al. 1998; Tacket et al. 1998, 2000; Thanavala et al. 2005). This dual functionality underscores the versatility of potato as both a biofactory and an oral delivery platform.

### 2.2 Tubers as Propagation Material for PMF

Vegetative propagation provides a major advantage for maintaining genetic fidelity in PMF systems. Since tuber‐based reproduction bypasses meiotic segregation, it ensures the generation of isogenic lines and reproducible production batches (De Wilde et al. 2002). Rapid multiplication of specific genotypes is possible in both field and in vitro settings, offering practical flexibility compared to seed‐propagated systems (Bradshaw 2021). By contrast, seed‐propagated hosts enable rapid, decentralised scale‐up from uniform seed lots once lines are fixed, a pace that clonal tuber multiplication cannot easily match (Buyel 2024).

Given the reliance on vegetative propagation, biosafety concerns related to transgene flow and environmental escape are comparatively limited in potato (Halterman et al. 2016). Pollen dispersal is limited to ~10 m, and a 20 m isolation distance effectively prevents gene flow to nearby fields (Conner and Dale 1996). While seed‐based systems excel in rapid scale‐up, potato holds the biosafety advantage: transgene dissemination via true potato seed is minimal, unlike in cross‐pollinating crops such as oilseed rape ( *Brassica napus* L. subsp. *napus*), where gene escape remains a significant issue (Liu et al. 2013).

At the same time, as potatoes are a food crop, the potential perception of GM contamination throughout the production chain needs to be acknowledged (Clark and Maselko 2020). This challenge can be addressed through enclosed cultivation systems, tightly controlled propagation methods, and the physical separation of PMF‐specific production streams from food supply chains, consistent with existing pharmaceutical PMF implementations that use dedicated nonfood hosts and physically isolated, controlled‐environment production systems. Such approaches are commonly discussed as part of biosafety and containment strategies in PMF (Clark and Maselko 2020; Stockdale and Millwood 2023).

In addition to regular tubers, potato can be propagated as greenhouse‐produced minitubers and fully in vitro microtubers (Bradshaw 2021; Kieu et al. 2021). These small storage organs enable clonal multiplication of engineered lines under tightly controlled conditions, accelerating multiplication and supporting contained proof‐of‐concept studies (De Wilde et al. 2002; Gautam et al. 2021; Mamiya et al. 2020; Park and Cheong 2002). Minitubers are already used in agriculture and could become a scalable feedstock for RPs production once workflows are optimised, whereas microtubers provide a standardised, biosafe platform for early‐stage PMF development (Bradshaw 2021; De Wilde et al. 2002; Mamiya et al. 2020; Park and Cheong 2002; Tremblay, Feng, et al. 2011). In controlled settings, both formats shorten production cycles and standardise propagation (Buyel 2024; Eidenberger et al. 2023; Schillberg and Finnern 2021).

## 3 Decline of Interest in Potato as a PMF Platform

The initial optimism surrounding potato‐based biofactories has gradually diminished due to a combination of general and crop‐specific challenges. Potato has struggled with relatively low RP yield, complex DSP, limited scalability compared to seed‐propagated crops and increasing competition from other platforms (Schillberg and Finnern 2021). Attempts to enhance its suitability as a PMF platform have remained limited (Kim et al. 2008; Tremblay, Diao, et al. 2011). Even with the advent of genome editing, applications in potato have so far focused on agronomic traits rather than PMF, and no studies have systematically explored genome editing to enhance potato as a biofactory (Karlsson et al. 2024; McLaughlin et al. 2025). These technological and regulatory barriers, together with societal concerns about GMOs, have further constrained investment and acceptance (Halterman et al. 2016; Buyel 2024).

In Europe specifically, a historically stringent GMO framework has reinforced this effect; only recently have EU institutions undertaken reform efforts to recalibrate rules for new genomic techniques, as reflected in the European Commission's  2021 study and the European Parliament's  2024 first‐reading position (European Commission 2021; European Parliament 2024). These ongoing negotiations resulted in a provisional political agreement reached in December 2025, aimed at establishing a revised regulatory framework combining existing GMO safeguards with differentiated pathways for certain NGT plant categories (Council of the EU 2025).

In contrast, regulatory approaches outside Europe illustrate alternative, more operational pathways for plant molecular farming. In the United States, regulatory oversight of genetically engineered plants (including those used in molecular farming) is embedded within a modular federal framework spanning USDA‐APHIS, FDA and EPA. Although no dedicated regulatory pathway specific to PMF exists, the availability of permit‐based authorisations and differentiated regulatory mechanisms has historically enabled clearer routes for greenhouse deployment and field trials than in the EU (USDA‐APHIS 2025; USDA 2026; FDA n.d.).

**FIGURE 2:**
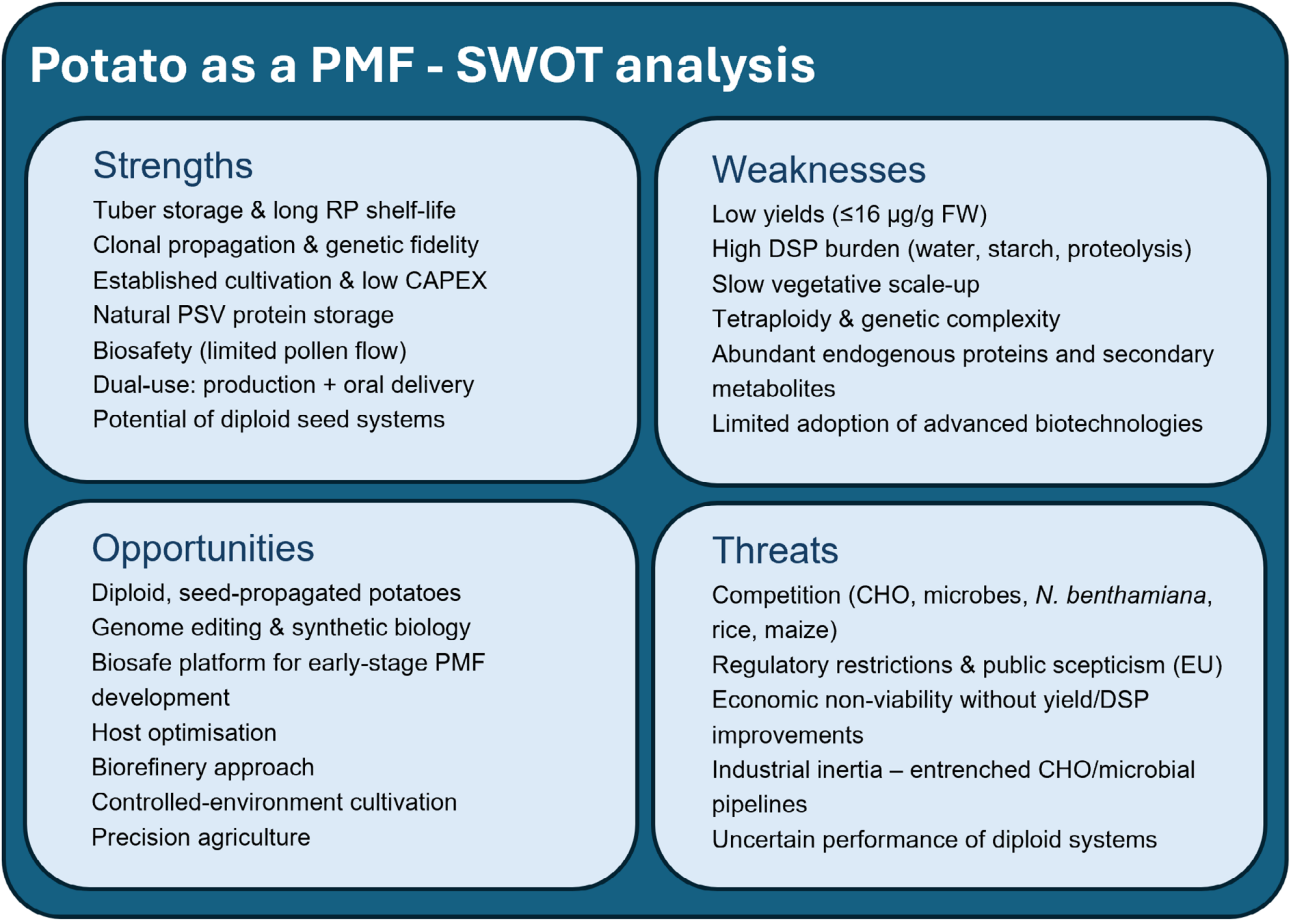
SWOT (Strengths, Weaknesses, Opportunities, Threats) analysis of potato as a platform for plant molecular farming. This analysis highlights both the unique storage advantages and the scalability/DSP bottlenecks that distinguish potato from other hosts.

To integrate the strengths and limitations of potato PMF, we provide a SWOT analysis (Figure 2), with key aspects elaborated in the sections of this review.

### 3.1 Comparative Performance of Platforms

*Nicotiana benthamiana* has become the dominant host for PMF, serving as a de facto standard due to its exceptional suitability for transient expression and rapid protein production, with quantitative yield ranges discussed later in this review. However, this dominance also reflects a focus on short‐term, high‐yield leaf‐based systems (Buyel 2026; Göritzer, Kallolimath, and Strasser 2025). In contrast, potato offers fundamentally different advantages, including stable expression, long‐term storage in tubers and decoupling production from cold‐chain logistics. These features position potato not as a competitor to *N. benthamiana*, but as a complementary platform for applications where storage organs and temporal separation of production and processing are critical.

As highlighted in the SWOT analysis, potato has been eclipsed by plant hosts that achieve higher expression levels and allow for simpler processing (Buyel 2024). In transient expression, *N. benthamiana* routinely reaches > 2 mg g^‐1^ FW of RPs, whereas stable transformed potato tubers rarely exceed ~0.016 mg g^‐1^ FW (De Wilde et al. 2002; Kogelmann et al. 2025; Richter et al. 2000; Tremblay, Feng, et al. 2011). Seed‐based platforms, such as maize and rice, provide favourable dry, starch‐based matrices that simplify harvest, storage and solids handling, lowering the per‐gram burden of DSP (Buyel 2024; Nandi et al. 2005; Schillberg et al. 2019). Oilseeds offer an additional DSP advantage when RPs are targeted to oil bodies via oleosin fusions, enabling simplified primary recovery (Board et al. 2022). In contrast, potato tissues contain a high proportion of water and starch, which reduces their suitability for storage prior to processing. Consequently, the recombinant target protein is present at lower concentrations, necessitating intensive dewatering and starch separation. These conditions elevate the risk of proteolysis and substantially increase the complexity and cost of DSP (De Wilde et al. 2002; Buyel 2024, 2026). A broader comparison of plant platforms, including technoeconomic aspects, is provided in Table 2.

**TABLE 2:** Comparative techno‐economic and biological parameters of representative expression systems for RPs production.

Parameter	Potato	Tobacco leaves	Tobacco BY2	*N. benthamiana*	Rice seeds	CHO Cells
Production site	tubers	leaves	cells in liquid culture	leaves	seeds	cells in liquid culture
Expression type	stable	stable	stable	transient	stable	stable
Representative RPs	MAK33 mAbs^1^	M12 mAbs^5^	M12 mAbs^7,17^	mAbs^8^ mAbs c4G7^15^	mAbs 2G12^18^ rhLF human lactoferrin^23^	mAbs^5,1,9^
RP Yield; mg/g FW, (pg/cell/day for liquid cell culture)	~0.008–0.016 mg/g FW^1,20^	~0.4 mg/g FW^5^	~0.05–0.07 mg/g FW^7^ (8 pg/cell/day)^17^	0.25–5 mg/g FW^8^	~0.037–0.046 mg/g rice flour^18^ ~5 mg/g dehusked rice grain^23^	50–90 pg/cell/day^5^(3 g/L)^19^
Batch cycle time	~6–10 weeks (minitubers); 4 months (full‐sized tubers)^1,9^	8 weeks^5^	1 week^7^	7–10 days^8,12,15^	6 months^23^	~2 weeks^19^
DSP cost share	90%–95%^1^	84%^5^	77%^7^	63%–65%^8^	90%–95%^23^	~35%^19^
Protein yield per batch	~1 g mAbs/100 kg FW^1^	~77 g mAbs/200 kg FW^5^	~4 g mAbs/200 L^5,7^	6.4 kg mAbs/9830 kg FW^8^	8 kg rhLF/180 kg dehusked grain^23^	17.4 kg mAbs/5800 L (16 × 10^6^ cells/mL)^19^
Water content of RPs‐producing tissue	~65%–86% ³	80%–90% ^10^	> 90%^17^	80%–90% ^12^ ^c^	~13% ^21^	60%‐80% ^13^
Time from transformation to obtaining stable propagation material	6–9 months (stably modified tubers)^1,4^	6–12 months (homozygous seeds for T‐DNA)^6^	~8 weeks^14^	37 days, ~6 week from seed to infiltration‐ready plants^12,16^	~9–12 months to homozygous transgenic seed (T1)^29^	~10–30 weeks depending on the method used^22^
Scalability^a^	++	+++	++/+++	++	+++	+++
Recovery	up to 25%^1^	88%^5^	75%–85%^7^	60%–80%^8^	96%‐97%^18^ 68%^23^	75%^19^
Possibility of time‐space separation of upstream and DSP processes^b^	++	+	+	+	+++	+
Susceptibility to infection with mammalian viruses and pathogens	none	none	none	none	none	yes
Availability of bioengineered hosts	Experimental only, e.g., Δpatatin ^11^	e.g ΔXT/FT^24^ ^d^	e.g ΔXT/FT^25^ ^d^	e.g., the NbXF‑KO^26^ NbXF‑KO^Gal^27^ ^d^	e.g., PhytoRice cell line^28^	Widely available; commercial glycoengineered lines

*Note:* References: ^1^De Wilde et al. 2002; ^2^Artsaenko et al. 1998; ^3^Bradshaw 2021; ^4^Craze et al. 2018; ^5^Schillberg et al. 2019; ^6^Fisher and Guiltinan 1995; ^7^Raven et al. 2015; ^8^Nandi et al. 2016; ^9^Santos and Rodriguez 2008; ^10^Bao and Wang 2016; ^11^Kim et al. 2019; ^12^Pruksarojanakul et al. 2025; ^13^general value for a mammalian cell, Neurohr and Amon 2020; ^14^Nocarova and Fischer 2009; ^15^Swope et al. 2022; ^16^Frigerio et al. 2022; ^17^Havenith et al. 2014; ^18^Vamvaka et al. 2016; ^19^Thaore et al. 2020; ^20^Richter et al. 2000; ^21^FAO (2010) standard for dry rice seeds; ^22^Zeh et al. 2024; ^23^Nandi et al. 2005; ^24^Göritzer et al. 2022; ^25^Herman et al. 2021; ^26^Kogelmann et al. 2024; ^27^Kogelmann et al. 2025; ^28^Shin et al. 2024; ^29^Tran et al. (2023).

^a^ Relative estimates for scalability and downstream‐process (DSP) separation potential: (+) low, (++) average, (+++) good scalability.

^b^ Relative estimates: (+) difficult, (++) partially possible, (+++) possible.

^c^ water content decreases significantly in the event of a hypersensitive response after agroinfiltration.

^d^ ΔXT/FT – double knockout line lacking xylosyltransferase (XT) and fucosyltransferase (FT) activities; NbXF‑KO – marker free *N.benthamiana* line ΔXT/FT; NbXF‐KO^Gal – NbXF‐KO line with chimeric β1,4‐galactosyltransferase targeted to the post‐Golgi.

### 3.2 Techno‐Economic Perspectives

For context, Table 2 also contrasts plant‐based systems with CHO cell culture as the current industrial benchmark. While detailed technoeconomic analyses (TEAs) are available for CHO as well as several plant systems, such assessments are lacking for potato (Nandi et al. 2005, 2016; Ridgley et al. 2023; Schillberg et al. 2019; Thaore et al. 2020). Nevertheless, proxy data indicate that approximately 1 g of monoclonal antibody can be recovered from ~100 kg of fresh tuber biomass (De Wilde et al. 2002). At field scale, assuming ~100 t harvested from ~2.5 ha, this equates to ~1 kg of purified antibody per cultivation cycle (De Wilde et al. 2002). While the technical feasibility of such output is evident, it falls significantly short of the requirements of commercial biomanufacturing. For comparison, optimised CHO cell lines routinely produce 50–90 pg cell^−1^ day^−1^ of IgG, with titers of 3–5 g L^−1^ and streamlined DSP pipelines, resulting in substantially lower costs per gram of product than potato or other plant platforms (Buyel 2024; Hansen et al. 2017; Kelley 2007).

In parallel, TEAs for various plant hosts have consistently highlighted that DSP contributes 60%–90% of total costs (Ridgley et al. 2023; Schillberg et al. 2019). By contrast, in CHO‐based production, DSP typically accounts for only ~30%–40% (Hansen et al. 2017; Kelley 2007), as high CAPEX and costly media inflate the upstream share. However, although CAPEX is markedly lower for plant‐based facilities, particularly those with standardised cultivation and harvest operations, than for mammalian cell culture (Holtz et al. 2015; McDonald and Holtz 2020), this advantage is insufficient to offset the disproportionately high DSP burden, and the very low RPs yields characteristic of potato.

A further critical technoeconomic limitation is scalability. Vegetative propagation via tubers enables rapid clonal multiplication of selected lines, but this method lacks the exponential scalability of seed‐based crops. In potato, a complete production cycle from transformation to harvest can take ~9 months (De Wilde et al. 2002). By contrast, although initial generation of homozygous *N. tabacum* lines requires 6–12 months (Fisher and Guiltinan 1995), once established, individual plants can yield millions of seeds, ensuring flexible and cost‐efficient scale‐up (Schillberg et al. 2019; Tremblay et al. 2010). Consequently, despite its short‐term propagation advantages, potato remains less scalable at industrial levels compared to seed‐based systems. For the conventional tetraploid, vegetatively propagated potato, these constraints remain valid (Bradshaw 2022). However, seed‐propagated diploid potato systems may represent an emerging exception (see ‘Unlocking the potential of diploid potato’).

In summary, the specific advantages of the potato, such as storage and vegetative propagation, are outweighed by low expression capacity, high processing costs and limited scalability. A direct comparison across expression hosts highlights these constraints (Table 2).

## 4 Optimisation Strategies for Potato‐Based PMF

Thirty‐five years after the first successful production of a human protein in potato (Sijmons et al. 1990), PMF has progressed from proof‐of‐concept to engineered design. Progress in expression‐cassette architecture, subcellular targeting and glycoengineering, genome editing and host‐chassis optimisation may help to overcome some of the earlier technical and biological limitations. Given the absence of a single plant host that has emerged as universally optimal for PMF (Buyel 2024; Schillberg et al. 2019), a rationally engineered potato line that has been developed to maximise yield, ensure expression stability and guarantee DSP compatibility deserves to be evaluated once again as a next‐generation biofactory.

### 4.1 Genetic Constructs and Synthetic Transgenes

In the early stages of research, attempts to produce RPs in potato occurred before the widespread adoption of codon optimisation, a practice that has since been demonstrated to enhance translational efficiency in heterologous systems (Webster et al. 2017; Fallahpour et al. 2025). Consequently, many early studies utilised native coding sequences, with plant‐optimised ORFs being adopted only sporadically. Notable exceptions to this include a plant‐codon–optimised gene encoding LT‐B antigen (Mason et al. 1998; Tacket et al. 1998) and a synthetic spidroin gene assembled from overlapping oligonucleotides that enabled silk‐protein accumulation in tubers (Scheller et al. 2001). The latter constitutes an early example of de novo gene synthesis for plant expression, anticipating current synthetic‐biology practices in construct design (Scheller et al. 2001).

At the time, construct design relied on strong constitutive or tuber‐active promoters: CaMV 35S (p35S often with a duplicated enhancer) and the patatin B33 (pB33), paired with conventional Agrobacterium‐derived terminators (De Wilde et al. 2002; Domansky et al. 1995; Ma et al. 1997; Rocha‐Sosa et al. 1989). Subsequent replacement of the terminators with stronger plant elements (t35S, 3′VSP, patatin terminator) increased accumulation across several targets (De Wilde et al. 2002; Mason et al. 1998; Ofoghi et al. 2000; Tacket et al. 1998). Potato‐derived regulatory sequences also proved broadly portable: Notably, the patatin pB33 promoter and the potato proteinase inhibitor II terminator (tPINII) function across multiple species, including monocots (Richter et al. 2000; Woodard et al. 2003). The tPINII was a key element in the first commercially relevant plant‐made RPs, bovine trypsin in maize (Woodard et al. 2003). Despite this cross‐species performance, these potato parts remain underused in potato PMF itself.

Precise expression control is essential for high‐yield, reliable RPs production and contributes to environmental biosafety by minimising unintended RPs accumulation and reducing the risk of gene flow or exposure beyond controlled settings (Eidenberger et al. 2023). While constitutive and tuber‐specific promoters dominate potato constructs, inducible or stimulus‐responsive systems remain largely unexplored. One example of such an approach is the ethanol‐inducible Bean Yellow Dwarf Virus (BeYDV) replicon platform, which enabled strong, transient, Rep‐mediated amplification of transgenes in potato leaves (Zhang and Mason 2006). Such systems can help mitigate toxicity problems that may arise during the production of some RPs in plant tissues, as illustrated by LT‐B in potato (Tacket et al. 1998), by restricting expression to defined developmental stages or environmental conditions. Among the few examples explored in potato, the B33 promoter, although tuber‐specific, is also inducible by sucrose in leaves (Rocha‐Sosa et al. 1989). This characteristic is advantageous for early testing of RPs production and construct efficiency in leaf tissue before tuberisation (De Wilde et al. 2002). The auxin‐responsive mas P2 promoter represents another inducible system, having been used to drive the accumulation of full‐length, bioactive human lactoferrin in potato tubers (Chong and Langridge 2000).

Beyond direct applications in PMF, potato has also served as a testing ground for innovative transcriptional tools. In potato specifically, orthogonal transcription via the Q‐system has boosted outputs from weak pathogen‐inducible promoters, enabling stand‐off detection of fluorescent reporters (Persad‐Russell et al. 2022). More recently, the pathogen‐inducible synthetic promoter 2xS‐4xD‐NpCABEcore was designed for potato, exemplifying the potential of synthetic promoters for tightly tunable, low‐background expression (Kauder et al. 2025). Beyond potatoes, advances across plants in minimal and synthetic promoter design (e.g., MinSyn libraries) enable graded expression and combinatorial tuning (Cai et al. 2020). Although optogenetic switches have so far been validated mainly in model species rather than in potato, they provide reversible, light‐controlled expression under plant‐compatible conditions and are, in principle, transferable to potato PMF (Konrad et al. 2023; Ochoa‐Fernández et al. 2020; Shikata and Denninger 2022). These advances, although not yet widely applied in PMF, broaden the regulatory toolkit available for future engineering of potato biofactories.

### 4.2 Targeting, Retention and Glycoengineering of RPs

The subcellular localisation of RPs critically determines their yield, folding, stability and post‐translational modifications (Göritzer, Melnik, et al. 2025). Early studies in potato demonstrated apoplastic secretion of human serum albumin (HSA) using SP enabling secretion (Sijmons et al. 1990), while other RPs were expressed without targeting sequences and remained in the cytoplasm, including Norwalk virus capsid protein (NVCP) (Mason et al. 1996) or glutamate decarboxylase GAD67 (Ma et al. 1997). Cytoplasmic localisation can be advantageous for certain proteins, such as VLP‐forming viral capsid proteins, but most cytoplasmic RPs are susceptible to proteolysis and lack essential post‐translational modifications, limiting the general applicability of this strategy (Ma et al. 1997; Mason et al. 1996). Importantly, the cytosol is a reducing environment that hampers the formation of disulfide bonds, making the correct folding of many secreted and structurally complex proteins difficult or impossible (Meyer et al. 2019).

To overcome these limitations, potato PMF has increasingly used ER retention, which provides a controlled intracellular environment for proper folding, basic N‐glycan formation and RPs stabilisation (Strasser 2023). Constructs combining N‐terminal SPs with C‐terminal retention motifs (KDEL, SEKDEL, DIKDEL) have markedly improved the accumulation of recombinant antibodies, antibody fragments and enzymes (Artsaenko et al. 1998; De Wilde et al. 2002; Chong and Langridge 2000; Mohammadi et al. 2020). By contrast, apoplast‐targeted expression, although enabling secretion and full glycosylation, resulted in lower yields. This has been demonstrated, for example, using SPs from potato proteinase inhibitor II (SP‐PINII) or Arabidopsis 2S albumin (SP‐At2S2), which successfully directed RPs to the apoplast but accumulated at lower levels compared with ER‐retained proteins (De Wilde et al. 2002; Farran et al. 2002). One factor contributing to the instability of secreted RPs, and thus to reduced yields, is the presence of poorly specific endogenous proteases along the secretory pathway, from the Golgi to the apoplast, which can drive RPs degradation in plants (Jutras et al. 2018). Mitigation strategies reported in PMF include co‐expression of apoplastic protease inhibitors and the development of targeted protease knockout or knockdown lines to reduce recombinant protein degradation along the secretory pathway (Goulet et al. 2012; Pillay et al. 2014; Mandal et al. 2014). In addition, the secretory pathway introduces plant‐type glycans, such as β(1,2)‐xylose and core α(1,3)‐fucose, during Golgi processing (Strasser 2023), which can further influence protein stability and overall yield. These plant‐type glycans may also compromise therapeutic efficacy or provoke immune responses in humans (Göritzer, Melnik, et al. 2025; Göritzer, Kallolimath, and Strasser 2025; Strasser 2023). Glycoengineering addresses these challenges by eliminating plant‐specific epitopes, generating human‐like, homogeneous N‐glycan profiles and enabling terminal sialylation, a modification critical for the half‐life and pharmacological performance of therapeutic glycoproteins. At the same time, it avoids nonhuman residues such as Neu5Gc, which are still present in some mammalian systems and may trigger immunogenic reactions (Kallolimath et al. 2016; Yehuda and Padler‐Karavani 2020). While such advanced N‐ and O‐glycoengineering approaches have been increasingly adopted in other plants (Frigerio et al. 2022; Göritzer et al. 2022; Shin et al. 2024; Strasser 2023; Uetz et al. 2024), they remain largely unexplored in potato. Further characterisation of the potato glycome, coupled with targeted editing, could therefore not only help align RPs glycosylation with human requirements but also enhance protein stability and thereby improve overall yields. For instance, in *N. benthamiana*, CRISPR/Cas modification of *CCT* genes combined with chaperone overexpression enhanced secretory IgA yields by an order of magnitude (Göritzer, Melnik, et al. 2025), highlighting a strategy that could, in principle, be adapted to potato‐based PMF platforms.

Although PSVs are the natural deposition site of endogenous storage proteins in potato tubers, deliberate targeting of RPs to this compartment has not yet been reported (Jørgensen et al. 2011; Marin Viegas et al. 2017). One notable exception in terms of localisation, albeit not of intentional targeting, is the nutritional protein AmA1, which accumulated predominantly in the cytoplasm and, to a lesser extent, in vacuoles of transgenic potato tubers (Chakraborty et al. 2000). The absence of systematic investigations nevertheless indicates a clear knowledge gap rather than a proven limitation. Evidence from other plant systems demonstrates both the opportunities and feasibility of this strategy (Marin Viegas et al. 2017). Vacuolar sorting signals (VSS) derived from potato proteinase inhibitor (PPI) enabled the accumulation of avidin and streptavidin in tobacco leaves, showing that potato‐derived motifs are functional and portable across species (Murray et al. 2002). The first FDA‐approved plant‐made pharmaceutical, taliglucerase alfa, relies on a C‐terminal VSS from tobacco chitinase A to direct glucocerebrosidase into carrot cell vacuoles, yielding mannose‐terminated glycans and eliminating the need for in vitro trimming (Shaaltiel et al. 2007). Recent reviews highlight the potential of PSV targeting, with soybean providing insights into vacuolar storage mechanisms (Vianna et al. 2023) and tobacco serving as a model for emerging subcellular engineering strategies (Song et al. 2024). Collectively, these examples reinforce that vacuolar deposition can offer a stable and biotechnologically relevant route for RPs accumulation. In potato, however, the vacuolar sorting machinery remains poorly characterised, and its systematic exploitation for PMF awaits further exploration. Unlocking this underexplored route will likely require the integration of genome editing, synthetic VSS design and multigene stacking within a rational, predictable framework.

### 4.3 Multigenic Stacking

Historically, potato PMF has relied predominantly on single‐gene T‐DNA cassettes, and coordinated multigene stacking beyond antibody heavy/light co‐expression has been uncommon in tubers (De Wilde et al. 2002). Two factors likely underlie this pattern: a relatively limited potato‐validated regulatory toolkit that discouraged complex multigene designs, and the cultivated potato tetraploidy, high heterozygosity and tetrasomic inheritance, which complicate transgene dosage control, stable co‐expression and breeding‐level fixation (Watanabe 2015; Bethke et al. 2022). Operationally, however, potato remains readily transformable (routine *Agrobacterium* transformation/regeneration), and vegetative propagation simplifies clonal maintenance. These features motivate the development of chassis and toolkits that enable reliable stacking in this host (Chincinska et al. 2023; Lian et al. 2025; Qu et al. 2024; Śliwka et al. 2025; Zhang et al. 2022). Nevertheless, progress is evident and stacking approaches have been successfully demonstrated in potato. For instance, the GAANTRY system enabled delivery of a ~10‐gene T‐DNA cassette with intact, single‐copy events (McCue et al. 2019), while other studies confirmed the feasibility of multigene stacking in this crop using alternative strategies (Jo et al. 2014; Ghislain et al. 2019). In parallel, modular multigene assembly platforms such as MIDAS‐P facilitate rapid, combinatorial construction of complex expression units that can be readily adapted to potato (Pinneh et al. 2022). Together with the widespread use of codon‐optimised ORFs and well‐characterised regulatory parts across PMF hosts (Chincinska et al. 2019; Coates et al. 2024), these developments provide a practical foundation for more systematic multigene engineering in potato, although broader adoption remains to be demonstrated (Rajput et al. 2025).

### 4.4 Host and Process Optimisation for RP Synthesis and Purification

Parallel to research demonstrating the potential of potato tubers as a source of RPs, several studies have explored ways to improve the host plant for use as a bioreactor. A significant line of investigation has focused on reducing the abundance of highly expressed native proteins to potentially facilitate DSP. Beyond their impact on downstream processing, the high abundance of storage proteins such as patatins may also interfere with RP accumulation by competing for cellular resources. Although direct experimental evidence in potato remains limited, observations from studies reducing patatin or starch content indirectly support the notion that host proteome composition can influence recombinant protein yield.

An early approach targeted the major tuber protein, patatin, using RNA interference (RNAi). By designing hairpin constructs against conserved motifs of patatin genes, Kim et al. (2008) achieved almost complete knockdown of patatin, while total soluble protein content remained comparable to the wild type (Kim et al. 2008). The authors hypothesised that a reduction in patatin levels could facilitate the purification of recombinant glycoproteins, with a particular focus on those intended for therapeutic use. However, the study did not directly test this hypothesis (Kim et al. 2008). In contrast, attempts to suppress the Kunitz protease inhibitor family were limited by extensive gene polymorphism, preventing uniform knockdown across all members (Speranskaya et al. 2012).

Building on the link between starch accumulation and patatin content, transgenic starch‐deficient plants were generated by inhibition of ADP‐glucose pyrophosphorylase, which reduced starch levels and concomitantly lowered patatin accumulation (Müller‐Röber et al. 1992). Similarly, RNAi‐mediated knockdown of the plastidial ATP/ADP transporter in transgenic potato increased total soluble protein content by ~50%. When a scFv antibody was expressed in these lines, its yield doubled compared to wild‐type controls, reaching 0.5% of TSP (Tremblay, Diao, et al. 2011). This represents one of the few instances where a genetically modified potato host was directly evaluated for RPs production.

Secondary metabolites, such as glycoalkaloids (GLAs), have been shown to interfere with both the recovery and consumption of tubers due to their toxicity. Silencing genes in the GLA biosynthetic pathway, including *GAME4* and 16α‐hydroxylase (*16DOX*), substantially reduced GLA levels without affecting plant growth (Itkin et al. 2013; Nakayasu et al. 2017, 2018). The CRISPR‐mediated knockout of *16DOX* resulted in the complete suppression of steroidal glycoalkaloid synthesis in tubers, thereby providing a more efficient background for the isolation of RPs. While these studies were chiefly concerned with reducing plant toxicity, they also demonstrate opportunities to optimise the host for RPs production.

In addition to these host‐modification strategies, newer genome editing technologies, including base editing and prime editing, offer highly precise alternatives to conventional knockouts (Perroud et al. 2022; Veillet et al. 2019). To date, however, their deployment in potato has been largely confined to agronomic and industrial traits such as disease resistance, stress tolerance, tuber quality or starch composition (Decima Oneto et al. 2025; Friberg et al. 2025; Karlsson et al. 2024; Massa et al. 2025). Nevertheless, the potential for PMF applications is considerable. Importantly, transcriptional control platforms such as CRISPRa/i, coupled with orthogonal circuits like the Q‐system, now allow programmable ON/OFF regulation of gene expression, for instance boosting weak promoters or restricting production to specific developmental stages (Persad‐Russell et al. 2022; Riabinina and Potter 2016; Zhang et al. 2024). In the context of PMF, such circuits could be adapted to induce RPs' expression only at harvest maturity or to silence contaminating proteins such as patatin or starch synthases, thereby increasing RP yield and purity.

Finally, DSP strategies complement host optimisation by improving RP recovery. For instance, a two‐step enzymatic process that starts with starch removal using α‐amylase (Termamyl; *Bacillus licheniformis* ) and is followed by cell‐wall depolymerisation with endo‐polygalacturonase M1 and endo‐β‐1,4‐galactanase increased protein recovery from ~47% to 63%–75% (Waglay et al. 2014, 2016). Multi‐enzymatic approaches have also been scaled from laboratory to pilot scale, with overall recoveries and the relative proportions of patatins versus other proteins depending on process conditions and feedstock (Waglay et al. 2019). Looking ahead, integrated enzyme cocktails together with modern clarification and continuous‐processing concepts, including membrane‐based clarification and (bio)flocculation‐supported filtration, are expected to further boost RP yield and scalability, though robust potato‐specific studies remain scarce (Buyel 2024, 2026).

In summary, the synergy between host optimisation, advanced genome editing circuits and modern DSP strategies could substantially enhance both yield and quality of RPs from potato tubers. At the same time, emerging precision agriculture tools provide a complementary opportunity to extend optimisation from the laboratory to the field. Hyperspectral imaging via unmanned aerial vehicle (UAV) or satellite platforms, supported by AI‐based prediction models, already enables accurate, noninvasive assessment of tuber yield, starch content and nutrient status before harvest (Alkhaled et al. 2025; Liu et al. 2025; Tatsumi and Usami 2024). Adapting such systems to stratify or preselect potato batches enriched in RPs or depleted of interfering metabolites would add a presorting layer that reduces DSP burden and increases overall process efficiency.

### 4.5 Unlocking the Potential of Diploid Potato for Next‐Generation Biofactories

Diploid potato clones offer a novel opportunity to reinvent the crop as a highly optimisable, genetically tractable biofactory (Lian et al. 2025; Smyda‐Dajmund et al. 2025; Zimnoch‐Guzowska and Flis 2021). Unlike cultivated tetraploid potato, diploid potato is more amenable to rapid genetic improvement, precise genome editing and hybrid breeding strategies (Lian et al. 2025). These characteristics, namely genetic tractability, stability and breeding flexibility, are of particular value when it comes to the rational design of plant hosts that are specifically tailored for PMF applications (Chincinska et al. 2023).

Consistent with the nucleotypic effect observed in plants, diploid potatoes are predicted to form smaller and more densely arranged cells compared with tetraploids (Pinto et al. 2024). Such cellular architecture could, in principle, favour more uniform transgene expression and facilitate metabolite exchange. Reduced cell size has been associated with increased productivity in microbial and mammalian bioreactor cultures (Buyel 2024; Guo et al. 2023). However, this relationship remains hypothetical in potato, and the link between cell size and RP yield should be regarded as a working hypothesis requiring direct experimental validation. Importantly, the diploid background aligns well with well‐established protoplast technologies, which provide a versatile platform for construct testing, CRISPR/Cas‐based genome editing, and even the generation of non‐GMO plants via transient delivery of DNA, RNA or RNP complexes. These tools can serve both as rapid proof‐of‐concept systems and as an entry point for regenerating precisely edited diploid lines, thereby complementing the advantages of diploid potatoes as biofactories (Carlsen et al. 2022; Friberg et al. 2025; Perroud et al. 2022; Yang et al. 2024).

The emergence of inbred diploid potato lines exhibiting high levels of homozygosity further strengthens the case for diploid systems in PMF. Such lines can facilitate genome editing, trait fixation, and experimental reproducibility to a greater extent than conventional tetraploid potatoes. The development of the Jan and mini‐Jan lines exemplifies this potential. Line Jan was derived from a cross between *Solanum tuberosum* group *Phureja* (DM1‐3) and the wild, self‐compatible diploid *Solanum chacoense* (M6), which carries the dominant *Sli* allele responsible for gametophytic self‐compatibility (Hosaka and Hanneman Jr 1998; Jansky et al. 2014). Successive generations of selfing and selection resulted in homozygous fixation of *Sli*, enabling reliable self‐pollination and high regeneration capacity (Xin et al. 2025). The mini‐Jan line was subsequently generated through CRISPR/Cas‐mediated mutation of the *ERECTA* gene in Jan, resulting in a stable dwarf phenotype that further demonstrates the amenability of these lines to precise genome engineering (Xin et al. 2025). From a PMF perspective, these lines can provide both a prototype for designing host potatoes and a conceptual model inspiring efforts to generate new biofactory lines through similar strategies.

At the same time, the broader challenge of self‐incompatibility (SI) in diploid potatoes illustrates why such breakthroughs are significant. For decades, gametophytic SI posed a barrier to the creation of inbred diploid lines. Recent advances in genomics have elucidated the genetic architecture of the S‐locus in *Solanum* spp., identifying numerous S‐RNase alleles and F‐box genes involved in pollen–pistil interactions (Jing et al. 2025; Lian et al. 2025). These findings pave the way for targeted manipulation of SI pathways, enabling controlled self‐pollination and the generation of elite, homozygous lines with desirable agronomic and biochemical traits (Ma et al. 2021).

Building on the milestones of potato inbred lines, production of F1 hybrid seeds can now be leveraged to combine heterotic alleles, improve yield stability and enhance RPs expression through precise genetic stacking. Seed propagation introduces a scalable, pathogen‐free multiplication system and allows for centralised production of uniform starting material, akin to microbial or mammalian cell banks. This could significantly reduce costs and enhance biosafety, especially in containment‐focused applications such as pharmaceutical production (Bethke et al. 2022; Buyel 2024; Halterman et al. 2016).

However, while the development and commercialisation of Jan and mini‐Jan have demonstrated technical feasibility, the broader adoption of diploid potato within PMF frameworks still requires careful validation under diverse agronomic and regulatory conditions (Xin et al. 2025). Despite their genetic tractability and experimental utility, diploid lines must still demonstrate clear superiority over conventional tetraploid cultivars in terms of agronomic performance, scalability, and cost‐efficiency. As Bethke et al. (2022) cautioned, ‘one must ask what the diploid system does that cannot already be accomplished with tetraploid lines’, highlighting the need for empirical, side‐by‐side comparisons that reflect real‐world use cases in molecular farming. This need is particularly pronounced, given that seed‐propagated diploid systems may introduce additional constraints, including inbreeding depression and agronomic trade‐offs, that could affect yield stability, tuber quality and large‐scale field deployment. Without such benchmarking, the long‐term industrial viability of diploid chassis remains speculative.

### 4.6 Alternative Plant Material and Potential Biofactory Chassis

Beyond diploid breeding systems, it is equally important to recognise that the potato belongs to a remarkably diverse *Solanum* gene pool. This diversity, represented by numerous cultivated and wild relatives, provides an almost inexhaustible reservoir of traits that could be harnessed to optimise biofactory performance (Bradshaw 2021; Kumar et al. 2025; Zhang et al. 2022).

Cultivated *S. tuberosum* exhibits comparatively restricted variation in protein composition across different cultivars, with tuber protein content generally ranging from 1.5%–2.5% of FW (Galdón et al. 2012; Lachman et al. 2005). Patatins constitute 17%–45% of total protein, and protease inhibitors 30%–50%, which can complicate RPs purification (Bártová et al. 2015). By contrast, other cultivated *Solanum* species, such as *S. phureja* and *S. andigenum*, exhibit broader protein diversity, with total protein levels of up to 8% DW and substantially lower patatin levels, along with enhanced resistance to biotic and abiotic stresses (Bártová et al. 2015; Kumar et al. 2025). These genotypes therefore represent a rich source of allelic variation and metabolic traits that could be leveraged to select or breed potato lines better suited for RPs production, before transgenic modifications are introduced. In this context, cisgenic approaches that exploit natural genetic variation, guided by genome‐wide association studies and re‐domestication strategies, offer promising opportunities for host optimisation (Azariadis et al. 2025; Lian et al. 2025; Śliwka et al. 2025; Zhang et al. 2022).

Together, these insights suggest a tiered strategy for expanding potato‐based PMF platforms. A first step lies in harnessing the natural allelic and metabolic diversity of diploid and wild potato relatives, including exploration of alternative *Solanum* germplasm with favourable cellular or biochemical traits that could enhance host performance. Building on this foundation, cisgenic and genome editing approaches can be applied to refine selected characteristics, such as protein composition or metabolite content, in a targeted manner. Finally, integrating alternative *Solanum* species or related tuberous crops as complementary biofactories would further diversify and strengthen the platform. This integrative framework maximises the potential of tuber‐based systems as PMF chassis by linking germplasm selection, targeted genetic refinement and experimental validation into a coherent development pipeline.

### 4.7 Towards a New‐Generation Bioreactor Potato

**FIGURE 3:**
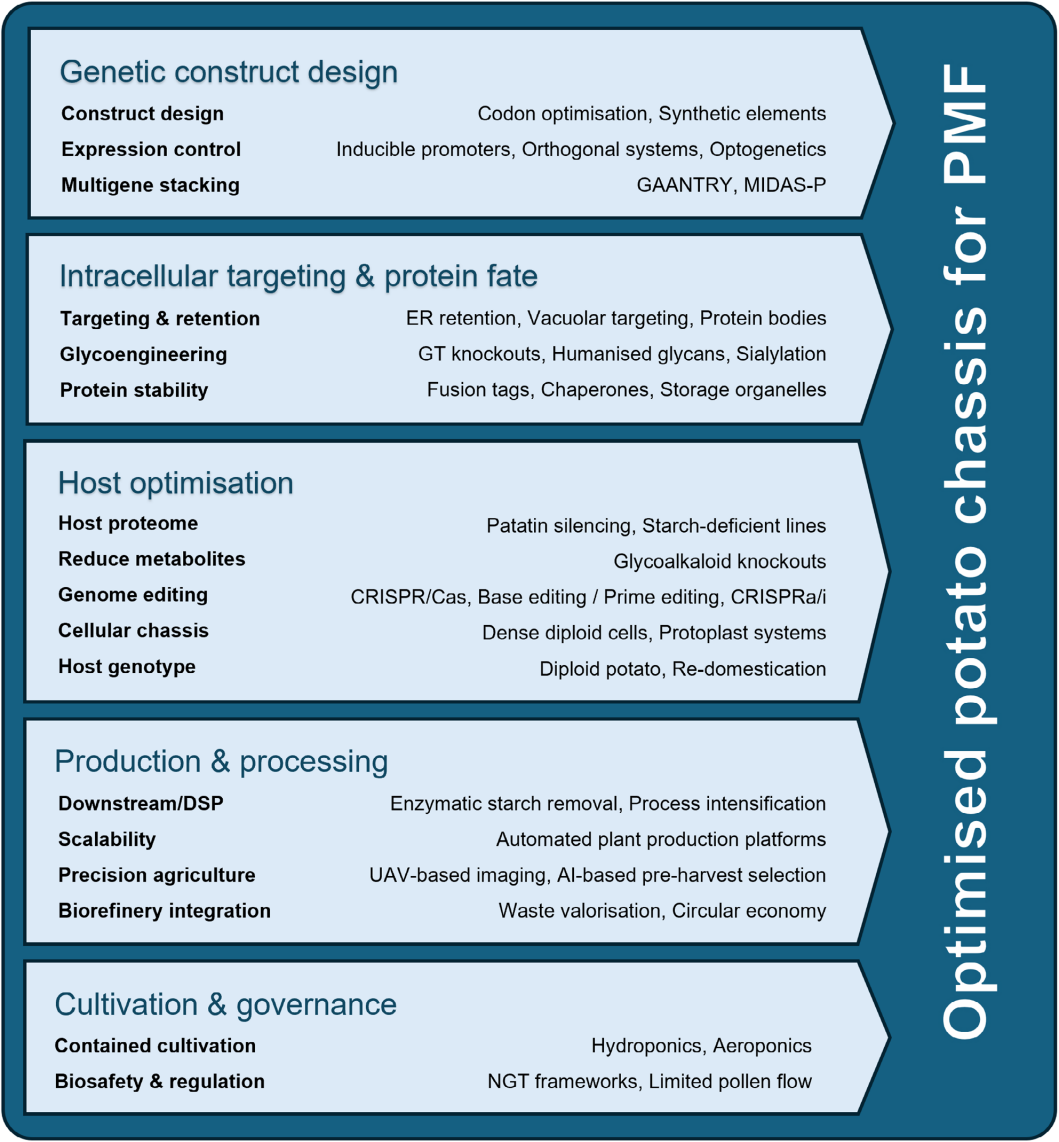
Schematic roadmap of technological strategies to enhance potato‐based molecular farming (conceptual illustration). Categories are organised hierarchically, from construct‐level optimisation through host engineering to DSP and regulatory aspects. Further explanations are provided in the main text. Abbreviations: GT, glycosyltransferase; UAV, unmanned aerial vehicles; NGTs, new genomic techniques.

The future of potato in PMF depends on its transformation into a purpose‐built biotech cultivar; a ‘bioreactor potato’ engineered from sequence to field deployment. The roadmap we propose (Figure 3) does not highlight isolated interventions, but a coordinated redesign that links construct engineering with intracellular protein routing, host optimisation, tailored processing and cultivation strategies. Its novelty lies in treating these layers as an integrated system rather than parallel options.

Realising this vision means moving beyond proof‐of‐concept: prioritising design rules that demonstrably increase yield and stability, adapting downstream pipelines to tuber biomass and embedding biosafety and governance from the outset. Only such integration can credibly position potato as a cost‐efficient, cold‐chain, independent biofactory for vaccines, biopharmaceuticals and industrial enzymes.

## 5 Perspectives: Potato as a Next‐Gen Integrated Biofactory

Building on this integrated roadmap, we now outline how potato PMF can expand beyond pharmaceuticals (Buyel 2024) into multifunctional, sustainable bioproduct pipelines (Bresnahan et al. 2025) that leverage native tuber biochemistry, biorefinery concepts and responsible innovation (Dietz and Muldoon‐Jacobs 2024).

### 5.1 Leveraging Native Tuber Biochemistry

Potato tubers are naturally endowed with a rich array of bioactive compounds (Biyimba et al. 2025; Friberg et al. 2025; Galves et al. 2023; Hu et al. 2024). The potential applications of potato‐derived biopolymers are numerous and span multiple domains. Some potato‐derived proteins, particularly in hydrolysed form, have already been evaluated for safety in cosmetic applications by the Cosmetic Ingredient Review (CIR 2017). Patatins exhibit emulsifying, texturizing, and neutral‐flavour characteristics, alongside lipase activity and antioxidant properties, which make them attractive for functional foods and dermocosmetic formulations (Lomolino et al. 2022). In addition to conventional protein isolates, potato protein microgels with adjustable interfacial properties have been developed, effectively stabilising emulsions and emphasising their potential applications in food, cosmetics and biomaterials (Akgonullu et al. 2024). Conversely, the efficacy of potato protease inhibitors, specifically PI2, has been examined in randomised clinical trials, demonstrating the capacity to regulate satiety and diminish postprandial appetite (Flechtner‐Mors et al. 2020). In turn, the potential of potato starch extends beyond its established role in the food industry. Ongoing research explores its use as a functional matrix for innovative biomaterials, cosmetic formulations and medical applications such as wound healing and drug delivery, where biodegradability and biocompatibility are key features (Biyimba et al. 2025; Lee and Hwang 2023; Lopes et al. 2023).

These examples emphasise the versatility and potential of potato‐derived molecules. Rather than diminishing these elements, their incorporation into PMF strategies may yield hybrid bio‐products that leverage the inherent characteristics of the tuber. In line with our earlier discussion of potato as an edible vaccine, it is worth considering tubers not only as vehicles for RPs but also as sources of complementary bioactive compounds that could support stability, uptake or efficacy. Early proof‐of‐concept studies with transgenic potatoes demonstrated the feasibility of oral immunisation (Haq et al. 1995; Mason et al. 1998; Tacket et al. 1998), and more recent perspectives highlight renewed interest in plant‐based delivery systems (Pudhuvai et al. 2025). Harnessing such synergies could reduce dependence on DSP, substantially lowering production costs while enhancing sustainability and societal acceptance of potato PMF systems.

### 5.2 Biorefinery Strategies for Sustainable Valorisation

A pivotal aspect of advancing PMF is the adoption of biorefinery approaches that transform all components of the potato plant into valuable products (Buyel 2024). Potato peel waste, a significant by‐product of industrial processing, is characterised by its high starch, fibre, and bioactive compound content. Through the implementation of bespoke valorisation strategies, these residues can be converted into biofuels, bioplastics, biosorbents and functional biomaterials, thereby minimising waste and promoting a circular bioeconomy. Such pathways are consistent with broader ‘zero‐waste’ principles and contribute to sustainable production chains (Ebrahimian et al. 2022; Rodríguez‐Martínez et al. 2023).

Beyond industrial applications, potato peels also represent a promising nutraceutical resource. Recent studies have emphasised the substantial health‐promoting potential of potato‐derived by‐products. A review of the literature reveals that potato peel extracts are rich in phenolics, flavonoids, and glycoalkaloids, which exhibit robust antioxidant and anti‐inflammatory activity (Hidayat et al. 2024; Vescovo et al. 2025). These observations have significant implications for the development of functional foods and nutraceuticals. The capacity of these by‐products to be utilised for industrial bioproducts, while concurrently functioning as a source of healthful ingredients, serves as a testament to their dual functionality.

An additional perspective relates to the current surge of research on plant‐associated microbiomes. One could envision potato bioreactors being coupled with symbiotic or engineered microbial consortia that not only support plant performance but also contribute to the efficient degradation of residual biomass after harvest. Recent work demonstrates that potato cultivars differ in their ability to recruit beneficial microbes (Martins et al. 2024), while soil microbial communities show strong metabolic capacities for recycling crop residues across seasons (Xie et al. 2025). Complementary reviews and experimental studies further highlight the potential of microbiome modulation in potato systems (Petrushin et al. 2024; Faist et al. 2023). Although still speculative, such approaches illustrate how PMF biorefineries might 1 day integrate biological alongside technological valorisation routes.

The integration of PMF operations with existing potato‐processing facilities could offer significant advantages. The integration of RPs production with starch or peel‐processing facilities would facilitate the cascading utilisation of residual biomass, thereby reducing logistical expenses and environmental impact (Buyel 2024; Gómez Palmero et al. 2025). This integration serves to reinforce the economic rationale for utilising potatoes as a biorefinery platform, while concomitantly aligning PMF with global sustainability objectives.

### 5.3 Enhancing Societal Trust Through Responsible Innovation

The success of PMF is contingent not only on technological innovation but also on societal acceptance. The transparent, environmentally responsible and socially beneficial design of PMF systems has the potential to foster public trust and facilitate the adoption of genome‐edited potatoes, a practice that varies across regions due to the influence of regulatory frameworks, cultural context, and risk perception (Bearth et al. 2024; Buyel 2024; Dietz and Muldoon‐Jacobs 2024; Halterman et al. 2016). By integrating public expectations and social values into research and development, PMF initiatives can enhance legitimacy and create opportunities for the responsible deployment of potato‐based biofactories (McCrea et al. 2024).

The utilisation of native tuber components within PMF systems has the potential to address public concerns regarding genetic modifications. By emphasising the utilisation of naturally occurring proteins and compounds, PMF will offer a more ‘natural’ and socially acceptable alternative to conventional pharmaceutical production. Emerging cultivation technologies, including hydroponic and aeroponic systems, have the potential to enable fully controlled, closed‐environment production of biotech potatoes, allowing precise regulation of light, temperature and nutrients (Kusnierek et al. 2023; Rajendran et al. 2024; Ritter et al. 2001). Such closed‐environment cultivation also provides a tangible barrier between genetically modified potatoes and the surrounding environment, directly addressing ecological and biosafety concerns. Although still in early development, these systems promise enhanced tuber yield, safety, reproducibility and public acceptance, particularly by alleviating apprehensions about field‐grown GM crops. Together, this illustrates that fully controlled, closed‐environment cultivation can offer a practical strategy to address ecological and societal concerns simultaneously (Halterman et al. 2016; Kusnierek et al. 2023; Ritter et al. 2001).

## 6 Conclusion

The future of potato‐based molecular farming lies in its transition from a narrowly focused pharmaceutical platform to a comprehensive, integrated biofactory. By harnessing the full potential of potato tubers, including their native biochemistry, functional by‐products and innovative cultivation strategies, PMF could help establish a biotechnological landscape that is sustainable, economically viable and socially accepted. When considered from this perspective, potatoes may serve as a model system for emerging biofactories, which are poised to merge high‐value production with environmental responsibility. This shift could also establish a platform for public engagement and education, thereby fostering trust in the development and implementation of novel agricultural technologies. The recent re‐emergence of industrial interest in potato indicates that its revival as a biofactory is no longer confined to academic discussion, but is now beginning to be actively explored at precommercial scale.

## Conflicts of Interest

The authors declare no conflicts of interest.

## Data Availability

Data sharing not applicable to this article as no datasets were generated or analysed during the current study.
